# Necrotising fasciitis in a patient treated with FOLFIRI-aflibercept for colorectal cancer: a case report

**DOI:** 10.1308/rcsann.2017.0143

**Published:** 2017-10-27

**Authors:** A Gonzaga-López, J Muñoz-Rodriguez, A Ruiz-Casado

**Affiliations:** ^1^Medical oncologist at Elda University Hospital; ^2^General surgeon at Puerta de Hierro Majadahonda University Hospital; ^3^Medical oncologist at Puerta de Hierro Majadahonda University Hospital

**Keywords:** Necrotising fasciitis, Colorectal neoplasms, Angiogenesis inhibitor, Bevacizumab, Aflibercept

## Abstract

Anti-angiogenics have become an important part of the treatment of several types of tumours such as ovarian, breast, lung and colorectal cancer. Necrotising fasciitis has been reported with bevacizumab but no cases have been reported with aflibercept, ramucirumab or regorafenib in patients with colorectal cancer. Necrotising fasciitis is a rare complication affecting one in 5000 bevacizumab users. We report the case of a 64-year-old man with stage IV rectosigmoid cancer under treatment with folinic acid, fluorouracil and irinotecan (FOLFIRI) and aflibercept, who developed a Fournier’s gangrene.

## Introduction

Colorectal cancer is one of the most frequent worldwide tumours. The treatment of advanced colorectal cancer has improved with the development of new drugs as vascular endothelial growth factor (VEGF) blockers and anti-epidermal growth factor receptor. The typical adverse effects of anti-angiogenics are hypertension, bleeding, gastrointestinal fistula and thromboembolic events. Anti-angiogenics have also been associated with an increased risk of infection but this toxicity is rarely fatal.^1,2^ We present the case of a 64-year-old man with stage IV rectosigmoid cancer diagnosed with Fournier’s gangrene, probably related to aflibercept.

## Case history

A 64-year-old man presented to the emergency department because of fever and buttock pain. He had a history of hypertension and hiatal hernia, and he was an ex-smoker and a mild drinker. Two years earlier, he had been diagnosed with stage IV *RAS* wild-type rectosigmoid adenocarcinoma with liver metastasis.

The patient underwent an open sigmoidectomy followed by FOLFOX6m as conversion chemotherapy . After four cycles with no response, second-line treatment with folinic acid, fluorouracil and irinotecan (FOLFIRI)-cetuximab was initiated. Despite achieving a tumoral response, the decision at the multidisciplinary team meeting was made for radiofrequency ablation because of macroscopic steatosis confirmed during the first surgical procedure. Further treatments included capecitabine and bevacizumab (AVEX) for seven cycles with stable disease and yttrium-90 radioembolisation of liver metastasis. Six months later, a colonoscopy revealed a local relapse at the rectal ampulla and liver progression. After 12 cycles, a new progression (rectal and liver) was confirmed. The patient had received one cycle of FOLFIRI-aflibercept 23 days before admission.

The patient presented to the emergency department with right buttock inflammation of 24 hours’ duration, fever up to 39 degrees celsius and chills. On examination, his vital signs revealed a blood pressure of 80/50 mmHg, tachycardia of 120 beats/minute and erythema, induration and a tender area in the right buttock. Analysis revealed leucocytosis 42.17 × 10E3/μl (reference range 4.0–11.5) with neutrophilia 83.7%, C reactive protein 211.70 mg/l (range 0.1–10), renal failure with creatinine 2.39 mg/dl (range 0.6–1.2) and lactate value 5.9 mmol/l. Creatinin kinase was 230 u/l (range 24–195). Coagulation disorder was also noted with prothrombin activity of 50%.

After a clinical diagnosis of Fournier’s gangrene, the surgeon performed an anal examination under anaesthesia and confirmed the diagnosis of necrotising fasciitis with perianal and right buttock involvement. An excisional debridement was performed. Blood and tissue cultures were collected and broad-spectrum empirical antibiotic treatment was initiated with meropenem, linezolid, metronidazole and fluconazole. The patient was admitted to intensive care and he underwent a further debridement after 24 hours of admission. Tissue cultures revealed polymicrobial infection with *Klebsiella pneumoniae*, *Proteus mirabilis*, *Clostridium beijerinckii* and *Candida albicans*. The evolution was satisfactory and he was discharged after 21 days to a rehabilitation hospital, where he remained for 3 months. The patient died 11 months after the diagnosis of Fournier’s gangrene.

## Discussion

Fournier’s gangrene is a type of necrotising fasciitis defined by deep-tissue destruction, including muscle fascia and subcutaneous fat localised at the perineum, gluteal muscles and, in advanced cases, affecting the abdominal wall. Traditional risk factors of necrotising fasciitis are diabetes, drug use, obesity, immunosuppression, recent surgery and traumatism. Clinical manifestations usually are acute and rapidly progressive oedema, erythema and tender area. Advanced cases are defined by toxic symptoms as fever, tachycardia and hypotension. Two types of necrotising fasciitis are described. Type I is a mixed infection caused by aerobic and anaerobic bacteria and type II is a monomicrobial infection caused typically by group A streptococcus or other beta-haemolytic streptococcus.

The mechanism of action of anti-VEGF drugs is inhibition of VEGF signalling by blocking VEGF ligand or VEGF receptor function and altering the tissue and tumor vascularization. Bevacizumab was the first anti-angiogenic drug approved by the US Food and Drug Administration (FDA) in 2004 for the treatment of colorectal cancer, associated to fluroropyrimidine-based chemotherapy, demonstrating an improvement in overall survival when used as first-line treatment. Later, in a phase III trial, aflibercept demonstrated a significant survival benefit associated with FOLFIRI in patients with metastatic colorectal cancer after treatment with an oxaliplatin-based chemotherapy.

In 2010, Gamboa *et al*. published the first case report of Fournier’s gangrene as a possible adverse effect of bevacizumab.^3^ A 67-year-old male with metastatic colorectal cancer treated with FOLFOX6m and bevacizumab developed a Fournier's gangrene, with no other risk factors apart from dyslipidaemia. In 2012, a report describing novel bevacizumab-related adverse events during FDA post-marketing surveillance was published.^4^ Necrotizing fasciitis was the described as a possible novel adverse event attributed to bevacizumab in 22 cases with an outcome of death in 13.6% of cases. The suggested mechanisms included subcutaneous artery thrombosis and tissue ischaemia, to which bevacizumab could contribute.

An increased risk of infections had been reported in several papers related to bevacizumab. Zhang *et al*.^1^ described a higher incidence of severe infections associated with aflibercept (7.3%) with a mortality of 2.2%. The risk of all-grade infections was much higher with aflibercept compared with bevacizumab (relative risk 4.07, *P* < 0.001).^1^

In our case, a 64-year-old man had developed a Fournier’s gangrene with the only known risk factor being immunosuppression related to chemotherapy. In addition, the patient was receiving treatment with aflibercept. Rectal relapse could be considered as another risk factor. To the best of our knowledge, this is the first reported case of Fournier’s gangrene in relation to an anti-angiogenic other than bevacizumab in colorectal cancer. In our opinion, necrotising fasciitis is a class adverse event related to anti-angiogenics.

Toxicity of oncological treatments is an important cause of hospital admission, morbidity and mortality. The quick diagnosis and the correct management of these complications is associated with a better outcome. The suspicion of necrotising fasciitis in a patient under treatment with anti-angiogenics is essential for prompt surgical treatment.

## Conclusion

This is the first case of necrotising fasciitis in a colorectal cancer patient possibly related to aflibercept treatment. It is important to suspect this entity in patients with recent treatment with anti-angiogenics because of the high mortality, morbidity and the need of prompt instigation of the proper treatment.
